# 4D strain analysis within non-infarcted myocardium of patients with ischemic cardiomyopathy: potential marker for the prediction of adverse cardiac events

**DOI:** 10.1186/1532-429X-18-S1-P233

**Published:** 2016-01-27

**Authors:** Alessandro Satriano, Nowell M Fine, Kate Fenwick, Dexter D Waters, Yoko Mikami, Haris Vaid, Derek V Exner, Carmen P Lydell, Andrew G Howarth, James A White, Bobak Heydari

**Affiliations:** 1 Stephenson Cardiac Imaging Centre, Calgary, AB Canada; 2 Division of Cardiology, School of Medicine, University of Calgary, Calgary, AB Canada; 3 Libin Cardiovascular Institute of Alberta, Calgary, AB Canada; 4 Queen's University, Kingston, AB Canada; 5 McGill University, Montréal, QC Canada; 6 University of Calgary, Calgary, AB Canada; 7 Department of Diagnostic Imaging, University of Calgary, Calgary, AB Canada

## Background

Adverse left ventricular remodeling following myocardial infarction (MI) remains one of the strongest prognostic indicators of adverse cardiac events. Increased wall stress and biomechanical strain within non-infarcted myocardium contribute to myocyte hypertrophy, fibrosis, and late adverse remodeling. This study aimed to demonstrate the feasibility of performing 4D strain analysis within non-infarcted myocardial segments through spatial registration to late gadolinium enhancement (LGE) imaging by cardiac magnetic resonance imaging (CMR) in ischemic cardiomyopathy (ICM) patients.

## Methods

Ninety-one patients from the ongoing prospective REFINE-ICD clinical trial underwent 1.5 Tesla CMR imaging (Avanto, Siemens, Germany) with multi-planar cine SSFP and LGE. Commercial software (cvi42, Circle Cardiovascular Inc.) was used to analyze LV volumes and perform segmental LGE analysis using a ≥3SD signal threshold. Segmental 4D strain analysis was incrementally performed using GIUSEPPE, an in-house software, with analysis restricted to those segments without LGE. In addition, 40 normal controls underwent identical 4D strain analysis. Inclusion criteria for the REFINE-ICD study includes adults with a history of prior myocardial infarction (3-15 months) and left ventricular ejection fraction (LVEF) between 36-50%.

## Results

**Figure 1:**
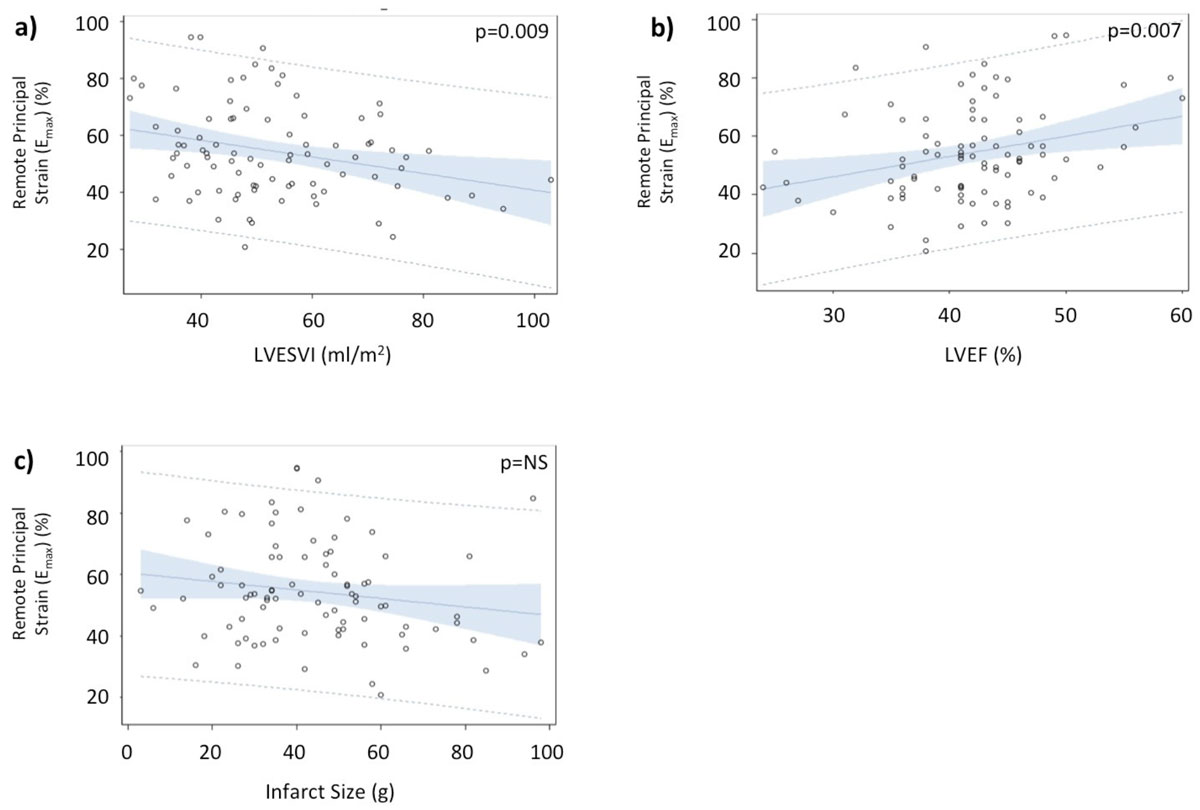
**Linear regression of Remote Maximum Principal Strain (E**_**max**_**) vs. LVESVI (a), LVEF (b) and Infarct Size (c) in patients affected by Ischemic Cardiomyopathy**.

Mean age of the ICM patients was 60.8 ± 9.7 years with mean LVEF of 41.7 ± 7.1%. The mean infarct size (percent of total myocardium) was 40.5 ± 12.7%. The findings of segmental 4D strain analysis are shown in Table 1 and Figure 1. Mean segmental principal, circumferential, and radial strain were significantly lower within non-infarcted myocardial segments compared to the myocardium of healthy controls. Linear regression analyses demonstrated a strong association between mean segmental principal strain and both LVEF and left-ventricular end-systolic volume. There was no association identified between observed strain values and total infarct size.

**Table 1:** Mean strain in normal patients and in remote segments of patients with ischemic cardiomyopathy.

Strain Variables	Normals	ICM (remote segments)	p-value
Maximum Principal Strain	74.76 ± 20.9	55.4 ± 18.9	<0.0001
Minimum Principal Strain	-20.04 ± 2.8	-18.04 ± 3.2	<0.001
Circumferential Strain	-15.01 ± 2.1	-11.59 ± 2.0	<0.0001
Radial Strain	56.32 ± 16.5	39.35 ± 16.6	<0.0001
Longitudinal Strain	-12.54 ± 2.3	-11.7 ± 2.6	0.08

## Conclusions

This study shows the feasibility of spatially matched 4D strain and LGE analysis to evaluate regional biomechanical strain of non-infarcted myocardial tissue in patients with an ischemic cardiomyopathy. Our results confirm impaired contractility relative to normal controls, independent of infarct size, and highlights the potential of remote tissue strain analysis to be evaluated as a prognostic marker for adverse cardiac events within this population.

